# The societal burden associated with adolescent idiopathic scoliosis: a cross-sectional burden-of-disease study

**DOI:** 10.1186/s12889-024-20423-x

**Published:** 2024-11-06

**Authors:** Thomáy-Claire Ayala Hoelen, Silvia M. A. A. Evers, Jacobus J. Arts, Paul C. Willems, Ghislaine A. P. G. van Mastrigt

**Affiliations:** 1 Department of Orthopedic Surgery, CAPHRI Research School, Maastricht University Medical Center (MUMC+), Maastricht, The Netherlands; 2 Department of Health Services Research, Faculty of Health, Medicine and Life Sciences, CAPHRI, Maastricht University, Maastricht, The Netherlands; 3 Trimbos Institute, Netherlands Institute of Mental Health and Addiction Utrecht, Utrecht, 3521 VS The Netherlands

**Keywords:** Adolescent idiopathic scoliosis, Burden of disease, Cost of illness, Dutch population, Health-related quality of life

## Abstract

**Background:**

In the general population the prevalence of adolescent idiopathic scoliosis (AIS) is 2–3%. There is growing awareness of how AIS affects the quality of life of patients. However, the extent of the societal burden AIS poses remains poorly understood. Therefore, this study aimed to determine the societal burden of AIS.

**Methods:**

A cross-sectional burden of disease study was conducted using a bottom-up, prevalence-based approach. Patients with AIS or guardians of a child diagnosed with AIS residing in the Netherlands were eligible for inclusion. The survey was distributed between June - December 2022 and was completed once by each participant. Costs were assessed using the institute for Medical Technology Assessment - Medical Consumption and Productivity Cost Questionnaires. The health-related quality of life (HRQoL) was assessed using the EuroQol 5D-5L/EuroQol 5D Youth and the Scoliosis Research Society-22 revised questionnaires. Costs and HRQoL were identified, measured, and valued.

**Results:**

Participants (*n* = 229) were predominantly female (92%), on average 35 years old, and were employed (65%). The societal cost for a patient with AIS in the Netherlands was €12,275 per year. The largest costs were estimated for the healthcare and productivity losses. The mean utility score for adults was 0.7 (SD 0.20). Severe pain was experienced by 10% of the adult participants and 44% reported to experience moderate pain/discomfort. Statistically significant differences between different age groups were present for the sector costs and HRQoL.

**Conclusions:**

AIS negatively impacts societal costs and the HRQoL. Reducing the burden that is posed on the productivity sector by AIS and further improving the HRQoL for AIS patients is needed.

**Supplementary Information:**

The online version contains supplementary material available at 10.1186/s12889-024-20423-x.

## Background

Adolescent idiopathic scoliosis (AIS) is a spinal deformity with unknown etiology and has a prevalence of 2–3% in the general population [1]. AIS occurs in adolescents (10–18 years old) and predominantly affects females [2]. In adolescents, the spinal deformity typically advances most in the midst of the growth spurt [3]. The burden AIS poses on health-related quality of life (HRQoL) aspects such as psychological well-being and physical functioning is increasingly recognized [1, 4, 5]. Patients with AIS are at an increased risk for mood disorders and often face significant challenges with the perception of their body image [6]. Sanders et al. found that 32% of the AIS patients suffer from significant psychological and emotional distress [7].

To help mitigate these effects and prevent curve progression conservative treatment can be started in mild to moderate spinal curves. Correction of the scoliotic curve achieved conservatively or surgically can alter aesthetic and thereby significantly improve individuals’ perception of their body [8–10]. More severe curves are best treated surgically to reduce serious health complications such as compromised pulmonary function, cardiovascular complications, persistent pain or psychological strain [11, 12]. Corrective spinal fusion is major invasive surgery with reported complication rates of 5–25% [13]. Furthermore, surgical correction is also considered a high-cost intervention [14–16].

Overall, patients with AIS utilize significant healthcare resources, with treatment costs increasing with the severity of symptoms [17]. Furthermore, AIS patients are at a higher risk of disability and unemployment if they have more severe clinical symptoms [18]. This presents an additional societal burden on an already overloaded healthcare system. To reduce the burden of AIS, researchers have suggested (institutionalized) screening by, e.g., school doctors to detect children with AIS earlier. The notion rests on the idea that early detection may help prevent progression and more severe symptoms [19]. Subsequently, it may be deduced that less invasive treatment with fewer costs will be incurred [19, 20]. However, controversy surrounding the benefits of early screening remains [21, 22].

Despite this growing body of evidence on the impact of AIS both on the patient and on the healthcare system [1, 4–7, 17, 18], to the best of our knowledge, there are no publications available that assess the total burden in terms of societal costs and HRQoL of AIS. Further understanding on the burden of AIS will help to attain the attention of policy and research agendas, thereby highlighting the need for more adequate preventive and treatment methods. Therefore, this cross-sectional study aimed to identify the resulting impact of AIS on patients residing in the Netherlands using a bottom-up, prevalence-based burden-of-disease study. As such the costs and generic HRQoL will be considered from a societal perspective.

## Methodology

### Study design

A societal perspective was adopted for this cross-sectional, prevalence-based, bottom-up approach cost-of-illness study. The study was conducted at one point in time [23] and considered the costs related to the total case volume over a fixed period (12 months). Since a societal perspective was adopted, all expenses, irrespective of who incurred them, were taken into account. Data was aggregated at the population level from two costing questionnaires and two questionnaires evaluating self-perceived HRQoL. The study was carried out at the Maastricht University Medical Centre (MUMC+). The Medical Ethical Testing Committee (METC) of the MUMC + ascertained that the study is not subject to the requirements of the Medical Research Involving Human Subjects Act (WMO) in the Netherlands (METC 2022–3166). Additionally, the MUMC + advisory board granted consent for the study. All participants signed an informed consent prior to the start of the study. To augment reporting quality and transparency, this study adhered to the Dutch standards for costing studies, the Consolidated Health Economic Evaluation Reporting Standards (CHEERS) and the guidelines published by Larg and Moss were taken into account [24–26]. Furthermore, a detailed study protocol was published prior to completion of the current study [27].

### Participants and procedure

Individuals diagnosed with AIS or guardians of a child diagnosed with AIS who were capable and willing to complete the questionnaire were eligible to join. Participants had to be residing in the Netherlands and able to read and write in the Dutch language. Based on prior research, a minimum of a 100 participants was aimed for to get sufficient variation in the patient population [28]. Patients were included successively between June and December 2022. Patients were approached by their treating physician and asked to complete the digital questionnaire. Additionally, the Dutch scoliosis patient society and foundation ‘’I love my back’’ were asked to disseminate the online questionnaire among their members.

### Data collection

The questionnaires started off with generic questions regarding patient demographics, followed by questions on costs and subsequently, questions on HRQoL (Appendix I-III). The online survey tool Qualtrics was used to distribute the questionnaires [29]. Completing the questionnaire required approximately 20–30 min.

### Cost-estimation

Three steps: identification, measurement and valuation, were utilized to calculate the costs associated with AIS following the bottom-up costing approach [23, 30].

### Identification

Cost categories facilitated the identification and organization of associated costs in line with the patient’s pathway. Health sector expenditures encompassed all costs associated with the diagnosis and management of AIS. These costs included costs for consultations, radiographs, prescribed medication and surgical procedures. Patient and family costs encompassed costs that were incurred by the patient or their family, e.g., expenses associated with traveling to and from the hospital. Additionally, productivity losses of the patient resulting from days off work or unemployment due to AIS were taken into consideration. Finally, expenses associated with missing schooldays were accounted for in the other sector category.

### Measurement

The institute for Medical Technology Assessment - Medical Consumption (iMTA-MCQ) and the Productivity Cost Questionnaire (iMTA-PCQ) were used to assess the costs associated with AIS [31]. The iMTA-MCQ and the iMTA-PCQ are generic instruments that measure medical consumption and productivity losses, respectively. The iMTA-MCQ accounts for a prior three-month period and contains 36 questions. The iMTA-PCQ comprises of 18 questions and is designed to evaluate various dimensions of productivity losses related to unpaid work such as absenteeism, presenteeism and productivity losses [32]. The questions in the iMTA-PCQ refer to a preceding 4-week period to minimize recall errors. Studies on the validity and reliability of both questionnaires are yet to be conducted.

### Valuation

Healthcare expenditure data was gathered from the iMTA-MCQ [31]. The Dutch standards for determining cost prices were utilized to determine cost values [25, 33, 34]. These costs consisted of prices for healthcare consultations, surgery and medication. Unit prices from the Dutch Central Bureau for Statistics were adjusted for inflation and indexed to the year 2022 were used [35]. Costs were presented in euros. To calculate the unit cost per item, the price per item was multiplied by the quantity of resource utilization [25]. The lowest unit price was used for medication, with the addition of costs for delivery in case of prescribed medication [33]. A default volume of one unit was applied, in case participants did not specify the number of appointments or consultations. Productivity losses were determined utilizing the friction cost method (FCM). By using the FCM, productivity losses are computed by multiplying the hours of absence from work by the standard employee rate, as described in the Dutch Costing Guidelines [25]. The FCM posits that replacement of an absent employee will occur after a 12-week friction period [25]. However, a friction period of 19.6 weeks was calculated to reflect the current labour market in the Netherlands. Informal care was valuated using the replacement cost method [25]. Despite the discrepancies in the recall periods of the administered questionnaires, all costing data were standardized to cover a three-month period and then extrapolated to 12 months.

### Self-perceived HRQoL assessment

#### Identification

The influence of AIS on the overall health and wellbeing of an individual was assessed using HRQoL questionnaires namely the EuroQol 5-dimensions (EQ-5D-5L) or EuroQol 5-dimensions Youth (EQ-5D-Y) and the Scoliosis Research Society-22 (SRS-22r) revised questionnaire [30, 36, 37].

#### Measurement

The EQ-5D-5L assesses the overall health condition of patients. This enables comparison between different populations and interventions, as the perspective is not limited to one specific disease [38]. Therefore, this questionnaire is often utilized and recommended for economic evaluations [23, 25]. The EQ-5D comprises five domains, each with a single question on: mobility, self-care, pain/discomfort, usual activities and anxiety/depression. Responses are rated on a 5-level scale ranging from: no problems to extreme problems. Additionally, the EQ-5D contains a visual analogue scale (EQ-VAS) for patients to self-report their health-status. The EQ-5D-5L has demonstrated acceptable psychometric properties making it a valid, reliable and responsive tool [39, 40]. Participants younger than 15 years old were given the EQ-5D-Y, a version of the EQ-5D-3L version that is tailored for children [41]. It shares the same five domains as the EQ-5D but uses a 3-level scale (no problems, some problems and a lot of problems).

A population specific HRQoL questionnaire was administered namely the SRS-22r questionnaire in conjunction with the EQ-5D-5L [42, 43]. Disease-specific questionnaires in general are tailored to specific patient populations, enabling measurement of HRQoL aspects not covered by generic questionnaires [38, 44]. For this reason these are less suitable for comparisons across diseases or populations [38]. The SRS-22r consists of 22 questions across five domains namely; function, pain, mental health, self-image and management satisfaction/dissatisfaction. Each domain consisting of five questions except for the fifth domain which consists of two questions. Responses are rated from 1 (worst) to 5 (best). A total score of 110 reflects the best HRQoL. Further details can be found in the study protocol [27].

#### Valuation

Summation of the answers to the EQ-5D questions results in 3125 health condition states [40]. Subsequently, these states were assigned a utility value, which was utilized in the economic assessment. The utility scores are country and version e.g. EQ-5D-5L [45] and EQ-5D-Y [46] specific. To examine the specific effects of AIS on the self-perceived HRQoL, the SRS-22r questionnaire was utilized. The SRS-22r captures aspects that are not covered by the generic EQ-5D-5L instrument.

### Data analyses

Non-parametric bootstrapping was performed using 1,000 replications for each cost category since cost data usually violates the normality assumption [23]. For the cost analyses an alpha level of 0.05 was set. To assess whether the HRQoL data was normally distributed, the Shapiro-Wilk test was used. A non-parametric test (Mann-Whitney U or Kruskal-Wallis in case of multiple groups) was performed when HRQoL data violated the normality assumption. Continuous data was described as mean (standard deviation) and categorical data was presented as count (percentage). Statistical significance was set at a p-value ≤ 0.05. Inconsistencies and completeness of data was assessed. Participants were excluded pairwise in case of missing data as it was assumed that these missing could be categorised as missing completely at random [47]. Analyses was performed using R-software version 4.0.3 (package: summarytools, forcats, dplyr, tableone, Eq. 5d).

### Sensitivity and subgroup analyses

Sensitivity analyses were conducted to evaluate how sensitive the results were to changes in methods or assumptions. A sensitivity analysis was performed by substituting the Dutch tariff with the UK and USA-tariff to test the methodological uncertainty. The minimally important difference (MID) of the EQ-5D-5L index score for Spain and England was reported to be 0.061 and 0.063,respectively [48]. Consequently, a threshold of 0.06 was used to assess the significance of differences in tariff between the Netherlands, UK and USA. Moreover, to assess the approaches used for determining productivity losses, the Human Capital Approach (HCA) was compared to the FCM approach [25]. Bootstrapped costs were estimated using the healthcare perspective as opposed to the societal perspective to analyse how alternative perspectives affects cost outcomes. Furthermore, the Kruskal-Wallis test and pairwise Wilcox test were used to determine whether there were significant differences between sub-groups based on age and gender.

## Results

### Patient characteristics

**Fig. 1:**
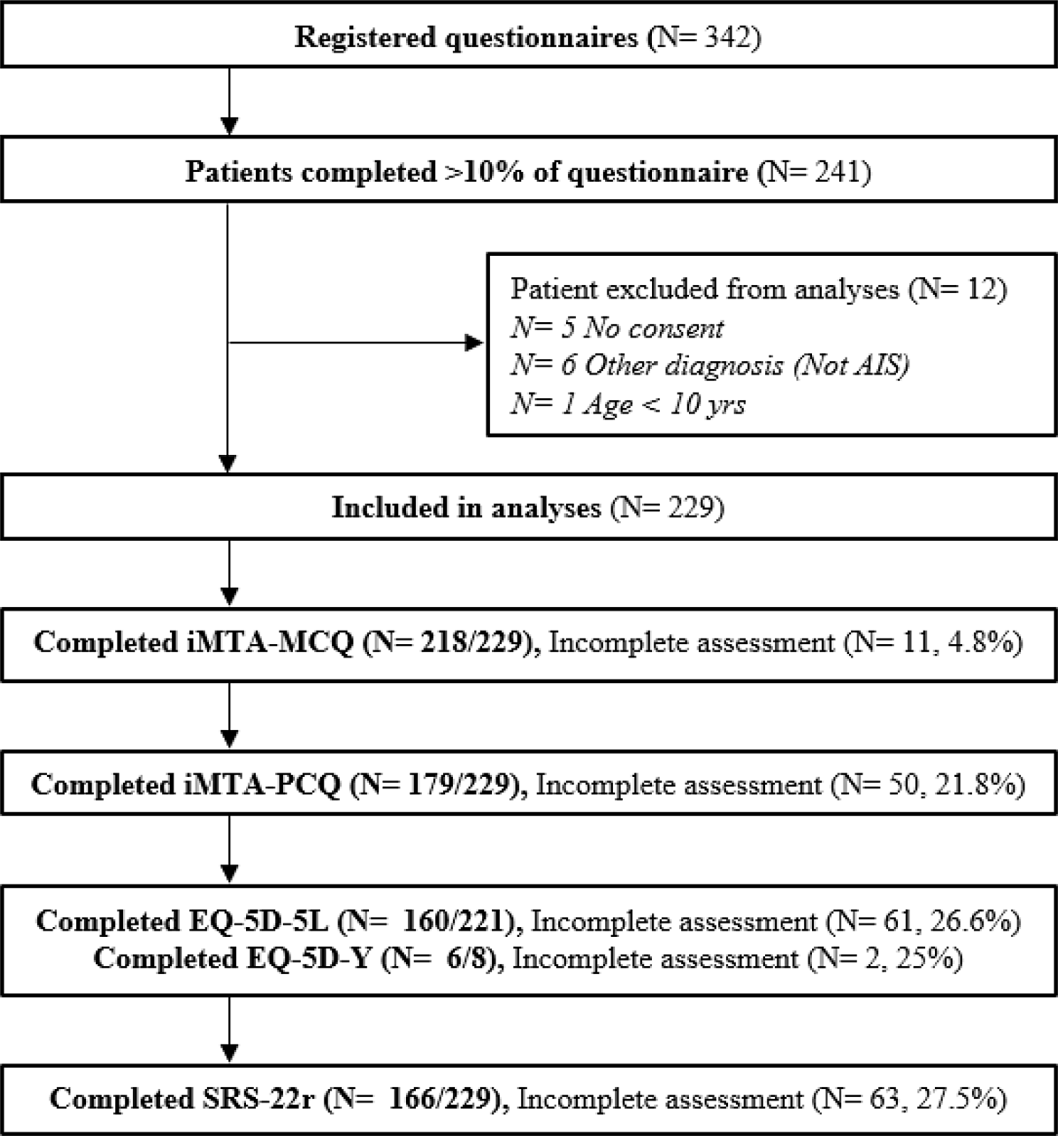
Flowchart inclusion participants and completeness of data

The online questionnaire was distributed from June to December 2022 and yielded 342 respondents. However, only 241 surveys were considered eligible for inclusion in the analysis. A 101 responses were excluded because they either provided no answers i.e. only opened the survey or completed less than 10% of the survey i.e. only indicating their willingness to participate and whether they were completing the questionnaire for themselves or their child. Of the 241 respondents, five respondents did not consent to take part in the study, six respondents were excluded since they were not diagnosed with AIS, and one was below the age of 10 and thus did not have the correct diagnosis. Subsequently, 229 participants were included in this study. Participants who completed the questionnaires (100%) were slightly older at 36.9 (SD 19.4) years compared to patients who only partially completed the questionnaires at 30.1 (SD 15.9) years, (*p* = 0.05). No statistically significant differences between the two groups were found for gender or paid employment. Further details on the completeness of data can be found in Fig. 1.

Of the 229 questionnaires, the majority was completed by respondents 16 years or older (*n* = 197, 86%), 24 questionnaires were completed by participants between the age of 12–15 years old (10.5%) and 8 (3.5%) questionnaires were filled in on behalf of someone else i.e., a parent of a child younger than 12 years old. Participants were predominantly female with an average age of 35.1 (SD 18.6) years. Most of the participants had an intermediate or higher level of education and approximately 65% had paid employment. Characteristics of patients who completed the questionnaire are presented in Table 1.

**Table 1:** Baseline characteristics people with adolescent idiopathic scoliosis, (*n* = 229)

Characteristics	*n* (%)
Gender	
Female	211 (92.1)
Male	17 (7.4)
Other	1 (0.4)
Age (yrs), mean (SD)	35.1 (18.6)
11–18	53 (23.1)
19–29	65 (28.4)
30–49	45 (19.7)
50+	66 (28.8)
Level of education^a^	
Lower level	30 (13.1)
Intermediate level of education	95 (41.9)
High level of education	93 (40.6)
Other	10 (4.4)
Currently in school/college	80 (34.9)
Work status	
Paid work	148 (64.6)
Unpaid work	81 (35.4)
Unemployed	5 (2.2)
Incapacitated	19 (8.3)
Pre-pension/pension	14 (6.1)

^a^Lower level of education defined as: no completed education, lower vocational education and intermediate level of education defined as: pre-vocational secondary education, senior secondary general education, pre-university education. High level of education defined as: higher professional education, university education

### Cost analyses

An overview of all items belonging to one of the four main categories e.g. healthcare sector costs, patient and family costs, other sector costs and productivity costs as well as the corresponding costs of these items are presented in Table 2.

**Table 2:** Societal costs in 2022 for people with adolescent idiopathic scoliosis

		Resource use: 3 months				Costs* (€), Mean (SD)	
Category	Unit	Unit Price	Min	Max	Mean (SD)	Time period: 3 months	12 months**
*Healthcare Sector Costs*							
General Practitioner	Consult	€40.3	0	20	1.3 (2.70)	€52.1 (108.80)	€208.3 (435.36)
Social Worker	Consult	€79.4	0	4	0.1 (0.51)	€8.8 (41.01)	€35.3 (164.03)
Physical therapist/ caesar therapist/ mensendieck/ manual therapist	Consult	€40.3	0	50	5.5 (7.08)	€222.3 (285.60)	€889.3 (1142.54)
Occupational therapist	Consult	€40.3	0	7	0.08 (0.65)	€3.2 (26.04)	€12.7 (104.15)
Speech therapist	Consult	€36.7	0	4	0.03 (0.29)	€1.02 (10.56)	€4.07 (42.22)
Dietician	Consult	€60.0	0	3	0.08 (0.38)	€5.0 (22.60)	€20.0 (90.39)
Homeopath/acupuncturist	Consult	€55.0	0	6	0.1 (0.66)	€7.1 (36.44)	€28.5 (145.75)
Psychologist, psychiatrist, psychotherapist	Consult	€107.2	0	12	0.7 (2.05)	€74.9 (220.20)	€299.7 (880.61)
Occupational physician	Consult	€100.0	0	2	0.1 (0.38)	€10.2 (38.43)	€40.7 (153.74)
Inpatient care (overnight hospital stay)	Days	€581.6	0	13	0.2 (1.14)	€88.9 (663.60)	€355.4 (2654.45)
Day treatment	Consult	€337.2	0	5	0.1 (0.51)	€46.8 (171.80)	€187.3 (687.35)
Outpatient care (less than 24 h)	Consult	€111.2	0	10	0.7 (1.31)	€74.6 (145.80)	€298.5 (583.14)
Emergency care (EHBO)	Consult	€316.4	0	1	0.02 (0.15)	€7.3 (47.69)	€29.3 (190.77)
Ambulance	Ride	€629.2	0	0	0	€0.0	€0.0
Medication prescription costs	Number	Variable	0	10	1.3 (1.87)	€231.8 (1345.79)	€927.1 (5383.17)
Home care- Household activities	Hours	€24.4	0	13	0.2 (1.49)	€4.8 (36.27)	€19.0 (145.07)
Home care- Personal care at home	Hours	€61.1	0	0	0	€0.0	€0.0
Home care- Nursing at home	Hours	€89.2	0	0	0	€0.0	€0.0
**Total Healthcare Sector Costs** ^**¶**^						**€849.05 (121.72)**	**€3**,**396.2 (486.87)**
*Patient & Family Costs*							
Transportation costs	Trip	Variable	0	50	7.7 (8.48)	€9.8 (10.69)	€39.07 (42.75)
Parking costs per visit at hospital	Trip	€3.7	0	11	0.9 (1.66)	€3.2 (6.11)	€12.7 (24.42)
Medication OTC costs	Number	Variable	0	2	0.5 (0.66)	€1.2 (5.89)	€4.9 (23.56)
**Total Patient & Family Costs** ^**¶**^						**€14.2 (1.16)**	**€56.7 (4.62)**
*Other Sector Costs*							
School days	Days	€46.0	0	30	2.5 (6.17)	€115.6 (283.96)	€461.9 (1135.84)
**Total Other Costs** ^**¶**^						**€30.2 (10.50)**	**€120.9 (42.00)**
*Productivity costs*							
Absenteeism	Working hours	€42.5	0	366	5.8 (39.00)	€639.6 (3,797.76)	€639.6 (3797.76)
Presenteeism	Working hours	€42.5	0	18	0.8 (1.90)	€97.04 (241.56)	€420.5 (1,046.77)
Productivity loss unpaid work	Hours	€17.1	0	180	8.0 (23.82)	€409.0 (1,221.74)	€1,772.1 (5,294.23)
**Total Productivity Costs** ^**¶**^						**€944.4 (248.64)**	**€2**,**319.9 (420.47)**
**Total Societal Costs** ^**¶**^						**€1**,**562.7 (270.95)**	**€12**,**274.5 (3**,**094.71)**

SD: standard deviation, OTC: over the counter drug

*All prices are indexed for the year 2022

**Prices are extrapolated to 12 months

^**¶**^Bootstrapped total costs

The total bootstrapped societal costs for a patient with AIS amounted to approximately €12,275 (SD €3,094.71) per year. The healthcare sector and productivity sector accounted for approximately 50% and 42% of the total societal costs, respectively (Appendix IV). The bootstrapped healthcare sector costs were estimated at just below €3400, - annually. The majority share was accounted for by prescribed medication costs, physical therapy treatments, inpatient and outpatient care as well as consultations for psychological well-being. A more detailed description of the prescribed and over the counter (OTC) medication used by the participants is presented in Appendix V. Costs for the productivity sector were estimated at €2,320 (SD 420.47) per year. Of the 229 participants included in this study 65% indicated to have paid work. Participants reported being absent from work on average 6 days every 4 weeks. Physical or mental complaints during work was experienced by 46% of the participants. This resulted in annual presenteeism costs being around €420,- (SD 1,046.77). Additionally, 45% of the participants experienced physical and/or mental complaints during unpaid work, which resulted in the loss of unpaid work to amount €1,772 (SD 5,294.23) per year. Furthermore, the estimated costs for the patient and family sector and other sectors were €57,- (SD 4.62) and €121,- (SD 42.00), respectively.

### HRQoL

The average utility score of the respondents (≥ 12 years old) was 0.71 (SD 0.2). The Dutch general population reference values are presented in Appendix VI, Table S2. Severe pain was experienced by 10% of the participants, moderate pain and/or discomfort was experienced by 44% and 30% of the participants indicated to experience slight pain and/discomfort. Additionally, 31% of the participants experienced moderate problems and 35% experienced slight problems whilst performing their usual activities. Furthermore, 41% of the respondents reported to have slight problems for the dimension anxiety/depression. Participants reported an average EQ-VAS score of 71.7 (SD 15.2, *n* = 159). The average score per domain as well as the number of participants that indicated having no problems, slight, moderate, severe or extreme problems per domain are presented in Table 3.

**Table 3:** EQ-5D, (*n* = 160)

Dimension	Mobility *n* (%)	Self-care *n* (%)	Usual activities *n* (%)	Pain/ discomfort *n* (%)	Anxiety/ depression *n* (%)
Level 1 (No problems)	87 (54.4)	139 (86.9)	46 (28.7)	24 (15.0)	65 (40.6)
Level 2 (Slight problems)	46 (28.7)	15 (9.4)	56 (35.0)	48 (30.0)	65 (40.6)
Level 3 (Moderate problems)	24 (15.0)	4 (2.5)	49 (30.6)	71 (44.4)	24 (15.0)
Level 4 (Severe problems)	3 (1.9)	2 (1.2)	9 (5.6)	16 (10.0)	6 (3.8)
Level 5 (Extreme problems/unable to do)	0 (0.0)	0 (0.0)	0 (0.0)	1 (0.6)	0 (0.0)

The average utility score of the six participants younger than 12 years old was 0.61 (SD 0.18). The average score per domain as well as the number of participants that indicated having no problems, some problems or severe problems per domain are presented in Appendix VI, Table S3. The average EQ_VAS score was 86.3 (SD 9.9).

The average scores per domain of the SRS-22r for the entire cohort are presented in Table 4. Although variation in average scores among the domains is minimal, the domain management (dis)satisfaction (3.0, SD 0.5) followed by the domain pain (3.0, SD 0.6) and the domain self-image (3.4, SD 0.7) scored the lowest. Overall, participants scored best on the domain of physical functioning. The mean score across all domains was 3.4 (SD 0.4) indicating a moderately good health-related quality of life.

**Table 4:** SRS-22r, (*n* = 166)

Domain	Mean*	SD	Range
Function	3.8	0.7	2.0–5.0
Pain	3.0	0.6	1.4–4.4
Mental health	3.6	0.7	1.6-5.0
Self-image	3.4	0.7	1.8-5.0
Management (dis)satisfaction	3.0	0.5	2.0-4.5
All domains	3.4	0.4	2.3–4.4

SD: standard deviation

*Mean score: 5 = best, 1 = worst

### Sensitivity analyses

Multiple sensitivity analyses were performed to assess how sensitive the results were to changes in methods or assumptions. As an alternative to the Dutch tariff, the UK tariff was used to calculate the total societal costs associated with AIS. Unlike when considering from a societal perspective, when considering the AIS burden from a healthcare perspective, only costs in the healthcare sector are considered. Thus, from a healthcare perspective the burden associated with AIS was estimated to be around €3400,- (SD 486.87) per year. Additionally, the HCA approach was used to determine the costs associated with productivity losses. When using the HCA approach is used the friction period is disregarded. The bootstrapped HCA productivity costs are estimated to be around €14,310 (SD €6,497) per individual with AIS per year. Furthermore, when considering the utility score of the respondents (≥ 12 years old) according to the Netherlands, UK and the USA value set an average utility score 0.87 (SD 0.17), 0.77 (SD 0.16) and 0.72 (SD 0.21) was found, respectively. In all cases, the average utility score reported by the participants in this study (0.71, SD 0.2) was lower than the average population utility scores. Additionally, the MID threshold of 0.06 has been met when comparing the utility score in this study by the average utility score of the Netherlands, UK and USA. This indicates that the change in utility score is important from a patient’s or clinician’s perspective.

### Subgroup analyses

To assess whether the burden of AIS varied for participants with different ages and gender, subgroup analyses were performed (Appendix VII, Table S4-S9). The individual sector costs differed significantly between the different age groups (Appendix VII, Table S4). However, the overall societal costs did not indicate any significant differences. The youngest age group (11–18 years old) differed significantly from the participants in the 30–49 years old group in all sectors. Costs calculated for the healthcare sector and patient and family sector were significantly higher for participants aged 11–18 years old compared to 19–29 year old participants whilst costs calculated for the productivity sector were significantly lower. Other sector costs were higher for participants between 11 and 18 years old compared to participants aged between 30 and 49 and 50+. Productivity costs were statistically significantly different between 11 and 18 and 50+, 19–29 and 30–49, 19–29 and 50 + year old participants, with 30–49 year old participants having the highest productivity costs. Furthermore, productivity costs were statistically significantly higher for females compared to males (Appendix VII, Table S5).

Subgroup analyses were performed to assess whether HRQoL outcomes differed between participants of different ages and gender. Statistically significant differences were found in the mean EQ-5D scores between participants in different age groups with 11–18 year old participants scoring lower on the domains: mobility (*p* < 0.01), usual activities (*p* < 0.01) and pain and discomfort (*p* < 0.01) and for the mean utility scores (*p* < 0.01) (Appendix VII, Table S6). When comparing EQ-5D scores of male and female participants, males were found to have significantly lower scores for the domains: usual activities (*p* < 0.05), pain and discomfort (*p* < 0.05), anxiety and depression (*p* < 0.05) and the utility scores (*p* < 0.05) (Appendix VII, Table S7). For the SRS-22r, statistically significant differences in age groups were found for the domains: function (*p* < 0.01), pain (*p* < 0.01), self-image (*p* = 0.01) and the overall score (*p* = 0.01) (Appendix VII, Table S8), with older participants scoring lower on the domains. No statistically significant differences were found when looking at the SRS-22r scores between male and female participants with AIS (Appendix VII, Table S9).

## Discussion

This burden of disease study evaluated the societal burden AIS poses in the Netherlands. The study findings indicate that the societal burden of AIS is estimated to be €12,275 per individual with AIS annually in the Netherlands. For AIS patients, the healthcare sector followed by the productivity costs are the largest contributors to the overall societal costs namely €3,396 and €2,320 per individual per year, respectively. These costs are also reflective of the physical and mental symptoms patients experience, with 10% of the participants indicating to experience severe pain/discomfort and 40% of the patients reporting to experience moderate pain/discomfort. The average EQ-5D-5L utility score of the participants in this study was 0.7 (SD 0.2) indicating that AIS has a significant impact on their health state.

An important finding is the variation in costs associated with different age groups. The costs for the healthcare sector and the patient & family sector were significantly lower for patients aged 19–29 years old compared to 11–18 years old. Notably, costs in these sectors increased for patients > 50 years old. This should be interpreted with caution since this increase may indicate a higher need for medical resources > 65 years of age in general [49], or could reflect the additional burden associated with managing AIS in older adults. It is crucial for future research to explore these age-related cost differences further to better understand the evolving needs of AIS patients over their lifespan.

When solely considering a healthcare perspective, the burden of AIS is significantly reduced compared to the societal perspective. The healthcare perspective excludes any costs other than direct costs associated with the provision of healthcare. However, since the second highest contribution to the burden is due to the productivity losses, solely the healthcare perspective would provide an underestimation of the true burden of AIS on society. With costs in the productivity sector accounting for over 40% of the total societal burden, the impact of AIS goes beyond costs made in the healthcare sector. Similar conclusions were reported in a study on the burden of overweight and obesity [28]. Hecker et al. reported a societal burden of approximately €11,500 per year for obesity, with the largest share of the costs accounted for by the productivity sector [28]. Additionally, Mastrigt et al. investigated the societal burden of stroke and reported that non-healthcare costs such as productivity losses and informal care significantly contribute to the total costs [50]. Furthermore, a large study on the economic cost of brain disorders also found that indirect costs such as absenteeism from work account for 40% of the costs [51]. Thus, more consideration for non-healthcare costs such as maintaining labour feasibility is essential and may deem more effective in reducing the overall societal costs compared to focussing primarily on reducing healthcare costs.

When comparing the utility score among patients with AIS (0.7) to the utility score for the general Dutch population (0.87 SD 0.17), it can be seen that AIS has a large impact on the experienced quality of life [45]. Hence, the MID in utility scores between AIS patients and the healthy Dutch population indicates that the threshold of 0.06 has been surpassed. The utility score derived from this study is slightly lower compared to previous studies looking at the HRQoL among AIS patients. Diarbakerli et al. measured HRQoL using the EQ-5D and reported utility scores of 0.82 for untreated patients, 0.82 for previously braced patients and 0.79 for surgically treated patients [52]. Larson et al. reported similar utility measures of 0.85 for untreated patients, 0.88 for patients in the bracing cohort and 0.83 for surgically treated patients [53]. Chua et al. reported slightly higher EQ-5D-5 L utility scores of 0.90 (SD 0.17) and 0.88 (SD 0.19) for patients treated with observation and bracing respectively [54]. Variations in utility scores may be due to differences in study design, country of study conduction as well as sample population. The study by Diarbakerli et al. performed in Sweden included patients with juvenile and adolescent idiopathic scoliosis whilst the study by Chua was conducted in Singapore. Although, the SRS-22r results showed minor differences between the domains it is interesting to note that patients scored lowest on the domains pain (3.0), management (dis)satisfaction (3.0) and self-image (3.4). These are important domains to consider since they can influence successful treatment outcomes. Although the average score for the domain management (dis)satisfaction varies among studies, it receives the worst scores when compared to the other domains [53–55]. Unlike previous publications, the average scores for the domain pain are relatively low e.g. 3.0 vs. scores > 4.0^52, 54^. However, our study includes AIS patients of all ages rather than patients aged between 11 and 21 years old and our subgroup analyses showed that pain worsens with age. Additionally, variations in pain perception and adaptation may be present due to factors such as cultural experience [56]. In contrast to the study findings of Diarbakerli et al. differences in SRS-22r scores for patients of different ages for the domains function, pain and self-image were found. Diarbakerli et al. included patients with juvenile and adolescent idiopathic scoliosis as well as comparing various treatment modalities which could have accounted for the differences in findings [52].

### Generalizability and transferability

This study may provide a template for burden of disease studies in alternative contexts. However, the question rises whether the study findings are generalizable across regions and countries. Country-specific analysis may provide the most accurate results, given the disparities in healthcare structure and funding across countries. Although the FCM is advised by the Dutch Costing Guidelines [25] the HCA was also performed for comparison since this is recommended in numerous other countries. Since the adoption of either method depends largely on subjective evaluation and preference, providing both estimates ensures transferability and generalizability [57]. The SRS-22r is a standardized, generally acknowledged questionnaire which allows for between-study comparisons. The EQ-5D is also standardized and internationally well recognized but the utility value sets are country specific making comparison more challenging.

### Implications

The findings of this study may be relevant to both health care and general policy, as well as clinical practice. Firstly, there is need for more education on the effects of scoliosis and the difficulties that patients may experience in their working environment. The societal impact of AIS is predominantly due to the healthcare and productivity sectors. In an already burdened labour market, more attention towards an ergonomic workplace may help to avoid presenteeism and absenteeism. Consequently, productivity costs can be reduced drastically. More qualitative research is needed to determine what problems patients with AIS experience in the workplace and how this affects their productivity and presenteeism at work. Furthermore, the main domains that patients identified as experiencing problems in were pain/discomfort, anxiety/depression and management/satisfaction with their healthcare trajectory. Pain management, mental well-being and the dissatisfaction with the healthcare trajectory is a point of concern and warrants further research since it can impact successful treatment outcome. More research is needed with regards to the age-related costs considering the societal costs presented are most likely an underestimation since the burden on family and caretakers such as the resulting productivity losses of family members could not be assessed. Additionally, more extensive research in the Netherlands and other countries will help gain a broader perspective of all aspects that impact AIS and allow for between country comparisons.

### Strengths and limitations

A considerable strength of this study lies in the standardization and clarity of the methodology. Additionally, research integrity was ensured by the use of the Dutch costing guideline, the Dutch value sets for EQ-5D utility, registration of the study protocol and the use of the methodological reporting CHEERS guidelines [24, 26]. Furthermore, the bottom-up method was used to determine the costing and quality of life measurements as stated by the Dutch Costing Guidelines [58, 59]. However, this study also has limitations. The survey did not allow for verification of the diagnosis AIS. However, the definition of AIS was extensively described in the questionnaire to help participants determine whether their diagnosis was AIS. Voluntary participation may induce selection bias, which may restrict generalizability. Additionally, there was incompleteness of data that could have induced bias and potentially could reduce generalizability e.g. 101 participants completed less than 10% of the survey. The most likely reasons for incompleteness of data was the extensiveness of the survey considering completing the questionnaires required a considerable amount of time. To deal with this, pairwise deletion was adopted since it was assumed that the missing data could be categorized as missing completely at random (MCAR) [47]. The mean utility score for the EQ-5D-Y was 0.61. However, this score is not very informative considering the lack of power due to the small sample size of *n* = 6. Also, due to time constraints, a prevalence-based approach was implemented, restricting cost estimates to a single year rather than throughout a lifetime, impacting the ability to assess costs and HRQoL over time. Another limitation was the potential for recall bias and errors in the responses due to the questionnaires being both retrospective and self-reported. For example, the SRS-22r refers to a recall period of 6 months in some questions but also contains questions which refer to the past week which may be answered with less recall error as a result [60]. Regardless, the SRS-22r questionnaire is specific to the studied population and useful for between-study comparisons. Furthermore, utilization of standardized questionnaires implied no further information could be provided through additional questioning.

## Conclusion

The estimated societal burden associated with AIS in the Netherlands was €12,275 per individual per year. The average EQ-5D utility score was 0.7 and patients indicated that AIS caused them pain and induced problems with daily functioning and self-image. This indicates that AIS has an impact on the societal costs and HRQoL. More than 90% of the societal impact can be accounted for by costs associated with healthcare related (50%) and productivity losses (42%). It is crucial to identify factors that prohibit patients with AIS to perform their work optimally and to reduce the number of days absent from work due to illness or complaints. Thus, to reduce this burden more political and clinical attention should be paid to the prevention of symptoms and limitations within the labour sector and patient-centred healthcare trajectories. Furthermore, attention should be paid to pain management and shared decision making to increase patient satisfaction with the adopted treatment.

## Electronic supplementary material

Below is the link to the electronic supplementary material.

Supplementary Material 1

## Data Availability

The datasets generated and/or analysed during the study are available from the corresponding author upon reasonable request. A data availability statement is provided below the funding statement.

## Abbreviations

AIS: Adolescent Idiopathic Scoliosis
CHEERS: Consolidated Health Economic Evaluation Reporting Standards
EQ-5D-5L: EuroQol 5-level
EQ-5D-Y: EuroQol 5-dimensions Youth
EQ-VAS: EuroQol Visual Analogue Scale
FCM: Friction Cost Method
HCA: Human Capital Approach
HRQoL: Health-related Quality of Life
ICC: Intraclass Correlation Coefficient
iMTA-MCQ: Institute for Medical Technology Assessment - Medical Consumption Questionnaire
iMTA-PCQ: Institute for Medical Technology Assessment – Productivity Cost Questionnaire
METC: Medical Ethical Testing Committee
MUMC+: Maastricht University Medical Centre
SRS-22r: Scoliosis Research Society-22 Revised Version

## References

[1] Negrini S, Donzelli S, Aulisa AG, Czaprowski D, Schreiber S, de Mauroy JC, et al. 2016 SOSORT guidelines: orthopaedic and rehabilitation treatment of idiopathic scoliosis during growth. Scoliosis Spinal Disord. 2018;13:1–48. 10.1186/s13013-017-0145-8 PMC5795289 29435499

[2] Lombardi G, Akoume M-Y, Colombini A, Moreau A, Banfi G. Biochemistry of adolescent idiopathic scoliosis. Adv Clin Chem. 2011;54:165–82. 21874761 10.1016/b978-0-12-387025-4.00007-8

[3] Yim AP, Yeung H-Y, Hung VW, Lee K-M, Lam T-P, Ng BK, et al. Abnormal skeletal growth patterns in adolescent idiopathic scoliosis—a longitudinal study until skeletal maturity. Spine. 2012;37(18):E1148–54. 22565390 10.1097/BRS.0b013e31825c036d

[4] Piątek E, Zawadzka D, Ostrowska B. Correlation between clinical condition of scoliosis and perception of one’s body image by girls with adolescent idiopathic scoliosis. Physiotherapy Q. 2018;26(3):34.

[5] Wang H, Tetteroo D, Arts J, Markopoulos P, Ito K. Quality of life of adolescent idiopathic scoliosis patients under brace treatment: a brief communication of literature review. Qual Life Res. 2021;30(3):703–11. 33098493 10.1007/s11136-020-02671-7 PMC7952337

[6] Gallant J-N, Morgan CD, Stoklosa JB, Gannon SR, Shannon CN, Bonfield CM. Psychosocial difficulties in adolescent idiopathic scoliosis: body image, eating behaviors, and mood disorders. World Neurosurg. 2018;116:421–32. e1. 29803063 10.1016/j.wneu.2018.05.104

[7] Sanders AE, Andras LM, Iantorno SE, Hamilton A, Choi PD, Skaggs DL. Clinically significant psychological and emotional distress in 32% of adolescent idiopathic scoliosis patients. Spine Deformity. 2018;6(4):435–40. 29886916 10.1016/j.jspd.2017.12.014

[8] Jain A, Marks MC, Kelly MP, Lenke LG, Errico TJ, Lonner BS, et al. Cost-utility analysis of operative versus nonoperative treatment of thoracic adolescent idiopathic scoliosis. Spine. 2019;44(5):309–17. 30475341 10.1097/BRS.0000000000002936

[9] Lonner BS, Brochin R, Lewis R, Vig KS, Kassin G, Castillo A, et al. Body image disturbance improvement after operative correction of adolescent idiopathic scoliosis. Spine Deformity. 2019;7:741–5. 31495474 10.1016/j.jspd.2018.12.005

[10] Duramaz A, Yılmaz S, Ziroğlu N, Duramaz BB, Kara T. The effect of deformity correction on psychiatric condition of the adolescent with adolescent idiopathic scoliosis. Eur Spine J. 2018;27(9):2233–40. 29802465 10.1007/s00586-018-5639-4

[11] Choi J-H, Oh E-G, Lee H-J. Comparisons of postural habits, body image, and peer attachment for adolescents with idiopathic scoliosis and healthy adolescents. Child Health Nurs Res. 2011;17(3):167–73.

[12] Kontodimopoulos N, Damianou K, Stamatopoulou E, Kalampokis A, Loukos I. Children’s and parents’ perspectives of health-related quality of life in newly diagnosed adolescent idiopathic scoliosis. J Orthop. 2018;15(2):319–23. 29556117 10.1016/j.jor.2018.02.003 PMC5856671

[13] Al-Mohrej OA, Aldakhil SS, Al-Rabiah MA, Al-Rabiah AM. Surgical treatment of adolescent idiopathic scoliosis: complications. Annals Med Surg. 2020;52:19–23. 10.1016/j.amsu.2020.02.004 PMC7052396 32153775

[14] Martin CT, Pugely AJ, Gao Y, Mendoza-Lattes SA, Ilgenfritz RM, Callaghan JJ, et al. Increasing hospital charges for adolescent idiopathic scoliosis in the United States. Spine. 2014;39(20):1676–82. 24983937 10.1097/BRS.0000000000000501

[15] Workman JK, Wilkes J, Presson AP, Xu Y, Heflin JA, Smith JT. Variation in adolescent idiopathic scoliosis surgery: implications for improving healthcare value. J Pediatr. 2018;195:213–9. e3. 29426688 10.1016/j.jpeds.2017.12.031

[16] Sarwahi V, Tran E, Vora R, Dowling TJ III, Galina J, Fakhoury J, et al. The volume-cost relationship: how does surgical volume affect cost and value in AIS surgery. Clin Spine Surg. 2022;35(9):E706–13. 35509023 10.1097/BSD.0000000000001338

[17] Glassman SD, Carreon LY, Shaffrey CI, Polly DW, Ondra SL, Berven SH, et al. The costs and benefits of nonoperative management for adult scoliosis. Spine. 2010;35(5):578–82. 20118843 10.1097/BRS.0b013e3181b0f2f8

[18] Glassman SD, Berven S, Kostuik J, Dimar JR, Horton WC, Bridwell K. Nonsurgical resource utilization in adult spinal deformity. Spine. 2006;31(8):941–7. 16622386 10.1097/01.brs.0000209318.32148.8b

[19] Adobor RD, Joranger P, Steen H, Navrud S, Brox JI. A health economic evaluation of screening and treatment in patients with adolescent idiopathic scoliosis. Scoliosis. 2014;9:1–10. 25601889 10.1186/s13013-014-0021-8 PMC4298059

[20] Hresko MT, Schwend RM, Hostin RA. Early detection of scoliosis—what the USPSTF I means for us. JAMA Pediatr. 2018;172(3):216–7. 29318255 10.1001/jamapediatrics.2017.5585

[21] Dunn J, Henrikson NB, Morrison CC, Blasi PR, Nguyen M, Lin JS. Screening for adolescent idiopathic scoliosis: evidence report and systematic review for the US preventive services task force. JAMA. 2018;319(2):173–87. 29318283 10.1001/jama.2017.11669 PMC13480065

[22] Deurloo J, Verkerk P. To screen or not to screen for adolescent idiopathic scoliosis? A review of the literature. Public Health. 2015;129(9):1267–72. 26296849 10.1016/j.puhe.2015.07.021

[23] Van den Boom NA, Van Den Hurk AA, Kalmet PH, Poeze M, Evers SM. Economic evaluations in fracture research an introduction with examples of foot fractures. Injury. 2022;53(3):895–903. 10.1016/j.injury.2022.01.013 35034777

[24] Husereau D, Drummond M, Augustovski F, de Bekker-Grob E, Briggs AH, Carswell C et al. Consolidated Health Economic evaluation reporting standards 2022 (CHEERS 2022) statement: updated reporting guidance for health economic evaluations. Int J Technol Assess Health Care. 2022;38(1). 10.1017/S0266462321001732 35007499

[25] Hakkaart-van Roijen L, Van der Linden N, Bouwmans C, Kanters T, Tan SS. Kostenhandleiding. Methodologie van kostenonderzoek en referentieprijzen voor economische evaluaties in de gezondheidszorg In opdracht van Zorginstituut Nederland Geactualiseerde versie. 2015.

[26] Larg A, Moss JR. Cost-of-illness studies. PharmacoEconomics. 2011;29(8):653–71. 21604822 10.2165/11588380-000000000-00000

[27] Hoelen T-CA, Willems PC, Arts JJ, van Mastrigt G, Evers S. The economic and societal burden associated with adolescent idiopathic scoliosis: a burden-of-disease study protocol. North Am Spine Soc J (NASSJ). 2023;14:100231. 10.1016/j.xnsj.2023.100231 PMC10333714 37440982

[28] Hecker J, Freijer K, Hiligsmann M, Evers SMAA. Burden of disease study of overweight and obesity; the societal impact in terms of cost-of-illness and health-related quality of life. BMC Public Health. 2022;22(1). 10.1186/s12889-021-12449-2 PMC8740868 34996413

[29] Tharp K, Landrum J, editors. Qualtrics Advanced Survey Software Tools. Indiana University Workshop in Methods. 2017.

[30] Dahham J, Rizk R, Hiligsmann M, Daccache C, Khoury SJ, Darwish H, et al. The economic and societal burden of multiple sclerosis on lebanese society: a cost-of-illness and quality of life study protocol. Expert Rev Pharmacoecon Outcomes Res. 2022;22(5):869–76. 10.1080/14737167.2022.2008242 34826264

[31] Bouwmans C, Hakkaart-van Roijen L, Koopmanschap M, Krol M, Severens H, Brouwer W. Manual iMTA medical cost questionnaire (iMCQ). Rotterdam: iMTA, Erasmus Universiteit Rotterdam; 2013.

[32] Bouwmans C, Krol M, Severens H, Koopmanschap M, Brouwer W, Hakkaart-van Roijen L. The iMTA productivity cost questionnaire: a standardized instrument for measuring and valuing health-related productivity losses. Value Health. 2015;18(6):753–8. 26409601 10.1016/j.jval.2015.05.009

[33] Nederland Z. Medicijnkosten. Medicijnkosten nl (2022, accessed October 20022). 2022.

[34] Nederland Z. Farmacotherapeutisch kompas. 2022.

[35] Centraal Bureau Statistiek C, Jaarmutatie. consumentenprijsindex; vanaf 1963: CBS; 2021 https://opendata.cbs.nl/statline/#/CBS/nl/dataset/70936ned/table

[36] Devlin NJ, Brooks R. EQ-5D and the EuroQol Group: past, Present and Future. Appl Health Econ Health Policy. 2017;15(2):127–37. 28194657 10.1007/s40258-017-0310-5 PMC5343080

[37] Schlösser TP, Stadhouder A, Schimmel JJ, Lehr AM, van der Heijden GJ, Castelein RM. Reliability and validity of the adapted Dutch version of the revised Scoliosis Research Society 22-item questionnaire. Spine J. 2014;14(8):1663–72. 24360746 10.1016/j.spinee.2013.09.046

[38] Patrick DL, Deyo RA. Generic and disease-specific measures in assessing health status and quality of life. Med Care. 1989;27(3 Suppl):S217–32. 2646490 10.1097/00005650-198903001-00018

[39] Feng YS, Kohlmann T, Janssen MF, Buchholz I. Psychometric properties of the EQ-5D-5L: a systematic review of the literature. Qual Life Res. 2021;30:647–73. 10.1007/s11136-020-02688-y PMC7952346 33284428

[40] Buchholz I, Janssen MF, Kohlmann T, Feng Y-S. A systematic review of studies comparing the measurement properties of the three-level and five-level versions of the EQ-5D. PharmacoEconomics. 2018;36(6):645–61. 29572719 10.1007/s40273-018-0642-5 PMC5954044

[41] Van Reenen M, Janssen B, Oppe M, Kreimeier S, Greiner W, Stolk E. EuroQol Research Foundation. EQ-5d-Y User Guide. 2020.

[42] Monticone M, Nava C, Leggero V, Rocca B, Salvaderi S, Ferrante S, et al. Measurement properties of translated versions of the Scoliosis Research Society-22 Patient Questionnaire, SRS-22: a systematic review. Qual Life Res. 2015;24(8):1981–98. 25682366 10.1007/s11136-015-0935-5

[43] Haher TR, Gorup JM, Shin TM, Homel P, Merola AA, Grogan DP, et al. Results of the Scoliosis Research Society instrument for evaluation of surgical outcome in adolescent idiopathic scoliosis. A multicenter study of 244 patients. Spine (Phila Pa 1976). 1999;24(14):1435–40. 10423788 10.1097/00007632-199907150-00008

[44] Peters M, Crocker H. Disease-Specific Questionnaire. In: Michalos AC, editor. Encyclopedia of Quality of Life and Well-Being Research. Dordrecht: Springer Netherlands; 2014. pp. 1667–8.

[45] Versteegh MM, Vermeulen KM, Evers SM, De Wit GA, Prenger R, Stolk EA. Dutch tariff for the five-level version of EQ-5D. Value Health. 2016;19(4):343–52. 27325326 10.1016/j.jval.2016.01.003

[46] Roudijk B, Sajjad A, Essers B, Lipman S, Stalmeier P, Finch AP. A value set for the EQ-5D-Y-3L in the Netherlands. Pharmacoeconomics. 2022;40(Suppl 2):193–203. 10.1007/s40273-022-01192-0 PMC9549846 36216977

[47] Allison PD. Missing Data. SAGE; 2001.

[48] McClure NS, Al Sayah F, Xie F, Luo N, Johnson JA. Instrument-defined estimates of the minimally important difference for EQ-5D-5L index scores. Value Health. 2017;20(4):644–50. 28408007 10.1016/j.jval.2016.11.015

[49] Bakx P, O’Donnell O, Van Doorslaer E. Spending on Health Care in the Netherlands: not going so Dutch. Fisc Stud. 2016;37(3–4):593–625.

[50] van Mastrigt G, van Heugten C, Visser-Meily A, Bremmers L, Evers S. Estimating the burden of stroke: two-year societal costs and generic health-related quality of life of the Restore4Stroke cohort. Int J Environ Res Public Health. 2022;19(17):11110. 36078828 10.3390/ijerph191711110 PMC9517815

[51] Olesen J, Gustavsson A, Svensson M, Wittchen HU, Jönsson B, Group CS, et al. The economic cost of brain disorders in Europe. Eur J Neurol. 2012;19(1):155–62. 22175760 10.1111/j.1468-1331.2011.03590.x

[52] Diarbakerli E, Grauers A, Danielsson A, Gerdhem P. Health-related quality of life in adulthood in untreated and treated individuals with adolescent or juvenile idiopathic scoliosis. JBJS. 2018;100(10):811–7. 10.2106/JBJS.17.00822 29762275

[53] Larson AN, Baky F, Ashraf A, Baghdadi YM, Treder V, Polly DW, et al. Minimum 20-year health-related quality of life and surgical rates after the treatment of adolescent idiopathic scoliosis. Spine Deformity. 2019;7(3):417–27. 31053312 10.1016/j.jspd.2018.09.003

[54] Chua YL, Toh AJN, Tan XYB, Pan DCY, Lee NKL, Lim KBL. Aspects of patient experience Associated with Improved Scoliosis Research Society-22 revised (SRS-22R) and European quality of life five-dimension five-level (EQ-5D-5L) scores in patients with adolescent idiopathic scoliosis Managed with Observation or Bracing. Spine. 2023;48(9):617–24. 36716381 10.1097/BRS.0000000000004585

[55] Cheung PWH, Wong CKH, Cheung JPY. An insight into the health-related quality of life of adolescent idiopathic scoliosis patients who are braced, observed, and previously braced. Spine. 2019;44(10):E596–605. 31046000 10.1097/BRS.0000000000002918

[56] Sharma S, Ferreira-Valente A, de Williams C, Abbott AC, Pais-Ribeiro JH, Jensen J. Group Differences between Countries and between Languages in Pain-related beliefs, coping, and Catastrophizing in Chronic Pain: a systematic review. Pain Med. 2020;21(9):1847–62. 32044980 10.1093/pm/pnz373 PMC7553014

[57] Krol M, Brouwer W. How to estimate productivity costs in economic evaluations. PharmacoEconomics. 2014;32(4):335–44. 24504850 10.1007/s40273-014-0132-3

[58] Tan SS, Rutten F, Van Ineveld B, Redekop W, Hakkaart-van Roijen L. Comparing methodologies for the cost estimation of hospital services. Eur J Health Econ. 2009;10(1):39–45. 18340472 10.1007/s10198-008-0101-x

[59] Wordsworth S, Ludbrook A. Comparing costing results in across country economic evaluations: the use of technology specific purchasing power parities. Health Econ. 2005;14(1):93–9. 15386663 10.1002/hec.913

[60] Clarke PM, Fiebig DG, Gerdtham U-G. Optimal recall length in survey design. J Health Econ. 2008;27(5):1275–84. 18667254 10.1016/j.jhealeco.2008.05.012

