# Supplementary material for: The societal burden associated with adolescent idiopathic scoliosis: a cross-sectional burden-of-disease study
Source: BMC Public Health. 2024 Nov 6;24:3065. doi: 10.1186/s12889-024-20423-x (PMC11539827; doi:10.1186/s12889-024-20423-x)
Supplement: Supplementary file 1 — Supplementary Material 1 [file 12889_2024_20423_MOESM1_ESM.pdf]

## Supplementary materials

I: iMTA Questionnaires

The iMTA-MCQ and iMTA-PCQ questionnaires are available upon request using:

*iMTA- Medical Consumption Questionnaire*

https://www.imta.nl/questionnaires/imcq/

*iMTA- Productivity Cost Questionnaire*

https://ww.imta.nl/questionnaires/ipcq/

II: EuroQol Questionnaires

EQ-5D-5L

Under each heading, please tick the ONE box that best describes your health TODAY.

**MOBILITY**

| | |
|---|---|
| I have no problems in walking about | ❑ |
| I have slight problems in walking about | ❑ |
| I have moderate problems in walking about | ❑ |
| I have severe problems in walking about | ❑ |
| I am unable to walk about | ❑ |

**SELF-CARE**

| | |
|---|---|
| I have no problems washing or dressing myself | ❑ |
| I have slight problems washing or dressing myself | ❑ |
| I have moderate problems washing or dressing myself | ❑ |
| I have severe problems washing or dressing myself | ❑ |
| I am unable to wash or dress myself | ❑ |

**USUAL ACTIVITIES** *(e.g. work, study, housework, family or leisure activities)*

| | |
|---|---|
| I have no problems doing my usual activities | ❑ |
| I have slight problems doing my usual activities | ❑ |
| I have moderate problems doing my usual activities | ❑ |
| I have severe problems doing my usual activities | ❑ |
| I am unable to do my usual activities | ❑ |

**PAIN / DISCOMFORT**

| | |
|---|---|
| I have no pain or discomfort | ❑ |
| I have slight pain or discomfort | ❑ |
| I have moderate pain or discomfort | ❑ |
| I have severe pain or discomfort | ❑ |
| I have extreme pain or discomfort | ❑ |

**ANXIETY / DEPRESSION**

| | |
|---|---|
| I am not anxious or depressed | ❑ |
| I am slightly anxious or depressed | ❑ |
| I am moderately anxious or depressed | ❑ |
| I am severely anxious or depressed | ❑ |
| I am extremely anxious or depressed | ❑ |

Sample

- We would like to know how good or bad your health is TODAY.
- This scale is numbered from 0 to 100.
- 100 means the best health you can imagine.
  0 means the worst health you can imagine.
- Please mark an X on the scale to indicate how your health is TODAY.
- Now, write the number you marked on the scale in the box below.

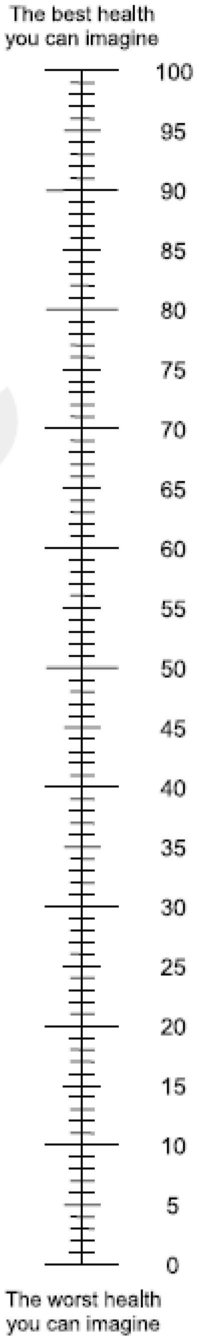


YOUR HEALTH TODAY = [ ]

Sample

EQ-5D-Y (EuroQol EQ-5D Youth)

Under each heading, please tick the ONE box that best describes your health TODAY.

**MOBILITY** *(walking about)*

- I have no problems walking about ☐
- I have some problems walking about ☐
- I have a lot of problems walking about ☐

**LOOKING AFTER MYSELF**

- I have no problems washing or dressing myself ☐
- I have some problems washing or dressing myself ☐
- I have a lot of problems washing or dressing myself ☐

**DOING USUAL ACTIVITIES** *(for example, going to school, hobbies, sports, playing, doing things with family or friends)*

- I have no problems doing my usual activities ☐
- I have some problems doing my usual activities ☐
- I have a lot of problems doing my usual activities ☐

**HAVING PAIN OR DISCOMFORT**

- I have no pain or discomfort ☐
- I have some pain or discomfort ☐
- I have a lot of pain or discomfort ☐

**FEELING WORRIED, SAD OR UNHAPPY**

- I am not worried, sad or unhappy ☐
- I am a bit worried, sad or unhappy ☐
- I am very worried, sad or unhappy ☐

Sample

- We would like to know how good or bad your health is TODAY.
- This line is numbered from 0 to 100.
- 100 means the best health you can imagine.
  0 means the worst health you can imagine.
- Please mark an X on the line that shows how your health is TODAY.
- Now, write the number you marked on the line in the box below.

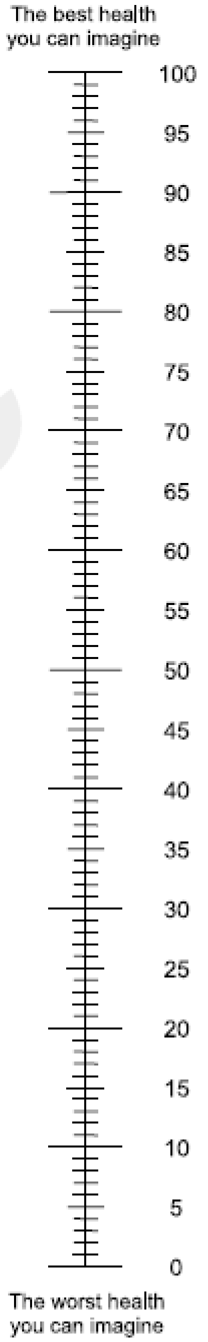


YOUR HEALTH TODAY = [ ]

Sample

**SRS-22r** Patient Questionnaire

Patient Name: ____________ ________ ______________ Date of Birth:
First MI Last Mo Day Yr

Today's Date: ______ ________ Age: --- +_______

Mo Day Yr Yrs Mo

Medical Record #: ____________________

**INSTRUCTIONS:** We are carefully evaluating the condition of your back and it is **IMPORTANT THAT YOU ANSWER EACH OF THESE QUESTIONS YOURSELF.** Please **CIRCLE THE ONE BEST ANSWER TO EACH QUESTION.**

1. Which one of the following best describes the amount of pain you have experienced during the past 6 months?

   None
   Mild
   Moderate
   Moderate to severe
   Severe

2. Which one of the following best describes the amount of pain you have experienced over the last month?

   None
   Mild
   Moderate
   Moderate to severe
   Severe

3. During the past 6 months have you been a very nervous person?

   None of the time
   A little of the time
   Some of the time
   Most of the time
   All of the time

**(CONTINUED ON NEXT PAGE)**

4. If you had to spend the rest of your life with your back shape as it is right now, how would you feel about it?

   Very happy
   Somewhat happy
   Neither happy nor unhappy
   Somewhat unhappy
   Very unhappy

5. What is your current level of activity?

   Bedridden
   Primarily no activity
   Light labor and light sports
   Moderate labor and moderate sports
   Full activities without restriction

6. How do you look in clothes?

   Very good
   Good
   Fair
   Bad
   Very bad

7. In the past 6 months have you felt so down in the dumps that nothing could cheer you up?

   Very often
   Often
   Sometimes
   Rarely
   Never

8. Do you experience back pain when at rest?

   Very often
   Often
   Sometimes
   Rarely
   Never

9. What is your current level of work/school activity?

   100% normal
   75% normal
   50% normal
   25% normal
   0% normal

**(CONTINUED ON NEXT PAGE)**

10. Which of the following best describes the appearance of your trunk; defined as the human body except for the head and extremities?

Very good
Good
Fair
Poor
Very Poor

11. Which one of the following best describes your pain medication use for back pain?

None
Non-narcotics weekly or less (e.g., aspirin, Tylenol, Ibuprofen)
Non-narcotics daily
Narcotics weekly or less (e.g. Tylenol III, Lorcet, Percocet)
Narcotics daily

12. Does your back limit your ability to do things around the house?

Never
Rarely
Sometimes
Often
Very Often

13. Have you felt calm and peaceful during the past 6 months?

All of the time
Most of the time
Some of the time
A little of the time
None of the time

14. Do you feel that your back condition affects your personal relationships?

None
Slightly
Mildly
Moderately
Severely

**(CONTINUED ON NEXT PAGE)**

15. Are you and/or your family experiencing financial difficulties because of your back?

Severely
Moderately
Mildly
Slightly
None

16. In the past 6 months have you felt down hearted and blue?

Never
Rarely
Sometimes
Often
Very often

17. In the last 3 months have you taken any days off of work, including household work, or school because of back pain?

0 days
1 day
2 days
3 days
4 or more days

18. Does your back condition limit your going out with friends/family?

Never
Rarely
Sometimes
Often
Very often

19. Do you feel attractive with your current back condition?

Yes, very
Yes, somewhat
Neither attractive nor unattractive
No, not very much
No, not at all

20. Have you been a happy person during the past 6 months?

None of the time
A little of the time
Some of the time
Most of the time
All of the time

**(CONTINUED ON NEXT PAGE)**

21. Are you satisfied with the results of your back management?

Very satisfied
Satisfied
Neither satisfied nor unsatisfied
Unsatisfied
Very unsatisfied

22. Would you have the same management again if you had the same condition?

Definitely yes
Probably yes
Not sure
Probably not
Definitely not

Thank you for completing this questionnaire. Please comment if you wish.

**END**

IV: Societal Costs and Percentage Share

*Table S1: Societal Costs and percentage share of total societal costs for patients with adolescent idiopathic scoliosis*

| **Costs*** | | **3 months** | **Share (%)** | | **12 months**** | **Share (%)** |
|---|---|---|---|---|---|---|
| **Total Healthcare Sector Costs** | € | 838,82 | 40% | € | 3.355,17 | 50% |
| **Total Patient & Family Costs** | € | 14,20 | 1% | € | 56,67 | 1% |
| **Total Other Costs** | € | 115,60 | 5% | € | 461,90 | 7% |
| **Total Productivity Costs** | € | 1.145,64 | 54% | € | 2.832,20 | 42% |
| **Total Societal Costs** | **€** | **2.114,26** | **100%** | **€** | **6.705,94** | **100%** |

*All prices are indexed for the year 2022

**Prices are extrapolated to 12 months

V: Drugs

OTC drugs

| Medication | Price per piece (€)/ dose | Source |
|---|---|---|
| Aspirine | €0,22 / 100 mg | 23 |
| Aspirine | €0,25 / 500 mg | 23 |
| Excedrin | €0,55 / 250/250/65mg | 23 |
| Ibuprofen | €0,06 /200 mg | 23 |
| ParLiquid | €0,28 /500 mg | 23 |
| Macrogol | €0,45 /10g | 23 |
| Naproxennatrium | €0,29 /220 mg | 23 |
| Nexium Control | €1,11 /20 mg | 23 |
| Panadol | €0,09 /500 mg | 23 |
| Paracetamol | €0,02 /500 mg | 23 |
| Reactine | €0,67 /10 mg | 23 |
| Rennie | €0,11 /680/80 mg | 23 |
| Riboflavine | €0,21 /10mg/ml | 23 |
| Saridon | €0,20 /150/250/50 mg | 23 |

Prescription drugs

| Medication | Price per piece (€)/ dose | Source |
|---|---|---|
| Acenocoumarol | €0,02 /1 mg | 23 |
| Acetylsalicylzuur | €0,03 /80 mg | 23 |
| Alendroninezuur | €0,25 /70 mg | 23 |
| Allopurinol | €0,05 /100 mg | 23 |
| Amlodipine | €0,02 /5 mg | 23 |
| Barnidipine | €0,71 /10 mg | 23 |
| Budeso/formo | €0,27 /400 ug | 23 |
| calci-boned3 orange | €0,13 /2,5 g | 23 |
| Candesartan | €0,03 /8 mg | 23 |
| CBD olie | €2,10 /100 mg/ml | 23 |
| CBD-olie+thc | €3,54 /20/13 mg/ml | 23 |
| Celecoxib | €0,09 /100 mg | 23 |
| Cetirizine | €0,03 /5 mg | 23 |
| Cholecalciferol | €0,12 /800 ie | 23 |
| Citalopram | €0,03 /20 mg | 23 |
| Clindamycine | €0,13 /10mg/g | 23 |
| Clonidine | €0,07 /0,15mg | 23 |
| Clopidogrel | €0,05 /75 mg | 23 |
| Cytomel | €1,32 /5ug | 23 |
| Desloratadine | €0,03 /5 mg | 23 |
| Diazepam | €0,03 /5 mg | 23 |
| Diclofenac | €0,03 /50 mg | 23 |
| Dymista | €16,87 / 137/50ug/do | 23 |
| Emselex | €0,88 /10 mg | 23 |
| Escitalopram | €0,04 /7,5 mg | 23 |
| Esomeprazol | €0,04 /20 mg | 23 |
| Estradiol pleister | €0,71 /50ug/24 uur | 23 |
| Etoricoxib | €0,09 /30 mg | 23 |

| | | |
|---|---|---|
| Euthyrox | €0,03 /25ug | 23 |
| Ezetimibe | €0,04 /10 mg | 23 |
| Ferrofumaraat | €0,03 /200 mg | 23 |
| Flixonase | €0,61 /1mg/ml | 23 |
| Flixotide | €1,47 /0,25mg/ml | 23 |
| Flixotide aer inhal | €9,15 /50ug/do | 23 |
| Fluticason | €0,12 /0,5 mg/g | 23 |
| Fluvoxamine | €0,10 /50mg | 23 |
| Foliumzuur | €0,04 /5 mg | 23 |
| Fosinopril | €0,08 /10 mg | 23 |
| Foster | €32,11 / 100/6ug/do | 23 |
| Fraxiparine inj. | €1,73 /9500ie/ml | 23 |
| Hydrochloorthiazide | €0,03 /25 mg | 23 |
| Hydrocobamine | €0,72 /500ug/ml | 23 |
| Letrozol | €0,13 /2,5 mg | 23 |
| Levothyroxine | €0,03 /25 ug | 23 |
| Liothyronine | €0,69 /25 ug | 23 |
| Lisdexamfetamine | €2,44 /20 mg | 23 |
| Losartan | €0,03 /25 mg | 23 |
| Lynestrenol | €0,13 /5 mg | 23 |
| Malarone | €2,11 /250/100 mg | 23 |
| Melatonine | €0,14 /1 mg | 23 |
| Meloxicam | €0,07 /7,5 mg | 23 |
| Metformine | €0,02 /500 mg | 23 |
| Methylfenidaat | €0,08 /5 mg | 23 |
| Metoprolol | €0,03 /50 mg | 23 |
| Mirtazapine | €0,05 /15 mg | 23 |
| Mometasonfuroaat | €4,69 /50 ug/do | 23 |
| Montelukast | €0,05 /10 mg | 23 |
| Movicolon poeder | €0,49 / sachet | 23 |
| Naproxen | €0,06 /250 mg | 23 |
| Nifedipine | €0,18 /60 mg | 23 |
| Nexium | €0,30 /20 mg | 23 |
| Nortrilen | €0,08 /10 mg | 23 |
| Nortriptyline | €0,06 /10 mg | 23 |
| Oculotect unidose | €0,18 /50 mg/ml | 23 |
| Omeprazol | €0,02 /20 mg | 23 |
| Ondansetron | €0,24 /4 mg | 23 |
| Oxazepam | €0,03 /10 mg | 23 |
| Oxycodon | €0,04 /5 mg | 23 |
| Pantoprazol | €0,02 /20 mg | 23 |
| Paroxetine | €0,06 /20 mg | 23 |
| Pravastatine | €0,07 /40 mg | 23 |
| Pregabaline | €0,06 /75 mg | 23 |
| Qvar | €9,70 /50 ug/do | 23 |
| Relvar | €31,41 / 92/22 ug/do | 23 |
| Rivotril | €0,04 /0,5 mg | 23 |
| Rizatriptan | €0,35 /10 mg | 23 |
| Rocaltrol | €0,27 /0,25 ug | 23 |
| Rosuvastatine | €0,02 /5 mg | 23 |
| Rupatadine | €0,07 /1 mg/ml | 23 |
| Salbutamol inhalator | €0,10 /200 ug | 23 |
| Sertraline | €0,03 /50 mg | 23 |

| | | |
|---|---|---|
| Sumatriptan | €0,25 /50 mg | 23 |
| Symbicort | €26,55 / 100/3 ug/do | 23 |
| Telmisartan | €0,04 /80 mg | 23 |
| Telmisartan | €0,05 /40 mg | 23 |
| Terbinafine | €0,09 /250 mg | 23 |
| Tiotropium inh | €0,89 /18 ug | 23 |
| Tirosint | €0,42 /13 ug | 23 |
| Tramadol | €0,05 /50 mg | 23 |
| Tramadol/Paracetamol | €0,10 /37,5/325 mg | 23 |
| Tranexaminezuur | €0,17 /50 mg/ml | 23 |
| Utrogestan | €0,19 /100 mg | 23 |
| Ventolin | €0,19 /1 mg/ml | 23 |
| VitamineD/Colecalciferol | €0,06 /800ie | 23 |
| Zaldiar | €0,15 / 37,5/325 mg | 23 |
| Zolpidemtartraat | €0,05 /10 mg | 23 |
| Zolpidemtartraat | €0,13 /5 mg | 23 |
| Zopiclon | €0,04 /7,5 mg | 23 |

## VI: EQ-5D

Table S2: Dutch general population EQ-5D-5L reference values

| Characteristics | Mean ± SD | Min. | Max. | N |
|---|---|---|---|---|
| **Age (y)** | | | | |
| **<20** | 0.958 ± 0.07 | 0.743 | 1 | 26 |
| **20 through** | 0.908 ± 0.146 | 0.031 | 1 | 158 |
| **30 through** | 0.903 ± 0.134 | 0.141 | 1 | 134 |
| **40 through** | 0.85 ± 0.196 | −0.16 | 1 | 202 |
| **50 through** | 0.857 ± 0.183 | −0.137 | 1 | 186 |
| **60 through** | 0.839 ± 0.179 | −0.003 | 1 | 158 |
| **70 and high** | 0.852 ± 0.148 | 0.335 | 1 | 106 |
| **Sex** | | | | |
| **Men** | 0.881 ± 0.172 | −0.012 | 1 | 480 |
| **Women** | 0.858 ± 0.168 | −0.16 | 1 | 497 |
| **Average** | 0.869 ± 0.170 | −0.16 | 1 | 979 |

EQ-5D-5L, EuroQol five-dimensional questionnaire five-level; Max., maximum; Min., minimum.

Reference Table S1: Versteegh, M. M., Vermeulen, K. M., Evers, S. M., De Wit, G. A., Prenger, R., & Stolk, E. A. (2016). Dutch tariff for the five-level version of EQ-5D. Value in health, 19(4), 343-352.

*Table S3: EQ-5D-Y, N=6*

| **Dimension** | **Mobility n(%)** | **Self-care n(%)** | **Usual activities n(%)** | **Pain/discomfort n(%)** | **Anxiety/ depression n(%)** |
|---|---|---|---|---|---|
| Level 1 (No problems) | 4 (66.7) | 6 (100) | 3 (50.0) | 1 (16.7) | 2 (33.3) |
| Level 2 (Some problems) | 2 (33.3) | 0 (0.0) | 3 (50.0) | 5 (83.3) | 4 (66.7) |
| Level 3 (Extreme problems) | 0 (0.0) | 0 (0.0) | 0 (0.0) | 0 (0.0) | 0 (0.0) |

Mean score: 1= no problems, 3= extreme problems

VAS: 86.3 (9.9)

VII: Subgroup analyses

Costs subgroup analyses

*Table S4: Societal and sector costs per age group*

| Sector | 11-18 yrs (n=50) | 19-29 yrs (n=58) | 30-49 yrs (n=43) | 50+ (n=65) | p-value* |
|---|---|---|---|---|---|
| Healthcare | €4909.43 | €2900.56 | €2761.73 | €3142.18 | **0.01** |
| Patient and family | €75.22 | €39.76 | €45.30 | €65.90 | **0.01** |
| Other | €287.23 | €214.28 | €0.00 | €0.00 | **<0.01** |
| Productivity | €172.69 | €457.61 | €4601.37 | €4127.19 | **<0.01** |
| Societal | €5444.57 | €3612.21 | €7408.39 | €7335.27 | 0.1 |

Wilcoxon rank Holm adjustment method multiple testing

Healthcare:

Sig. 11-18 age group and 19-29, 11-18 and 30-49

Patient and family:

Sig. 11-18 and 19-29, 11-18 and 30-49

Other sector:

Sig. 11-18 and 30-49, 11-18 and 30-49, 11-18 and 50+

Productivity:

Sig. 11-18 and 19-29, 11-18 and 30-49, 11-18 and 50+, 19-29 and 30-49, 19-29 and 50+

*Table S5: Societal and sector costs stratified for gender*

| Sector | Male (n=17) | Female (n=199) | p-value* |
|---|---|---|---|
| Healthcare | €3753.37 | €3381.37 | 0.5 |
| Patient and family | €63.83 | €56.35 | 0.4 |
| Other | €189.53 | €118.43 | 0.8 |
| Productivity | €117.22 | €2509.09 | **0.02** |
| Societal | €4123.95 | €6065.24 | 0.7 |

EQ-5D subgroup analyses

*Table S6: EQ-5D scores per age subgroup*

| **Domain** | **11-18 yrs (n=31)** | **19-29 yrs (n=41)** | **30-49 yrs (n=30)** | **50+ (n=57)** | **p-value*** |
|---|---|---|---|---|---|
| MO | 1.2 (0.48) | 1.3 (0.52) | 1.5 (0.63) | 2.2 (0.88) | **<0.01** |
| SC | 1.0 (0.18) | 1.1 (0.37) | 1.3 (0.79) | 1.3 (0.55) | 0.06 |
| UA | 1.5 (0.63) | 1.9 (0.86) | 2.3 (0.88) | 2.5 (0.83) | **<0.01** |
| PD | 1.9 (0.88) | 2.3 (0.82) | 2.8 (0.90) | 2.9 (0.72) | **<0.01** |
| AD | 1.7 (0.91) | 2.0 (0.80) | 1.9 (0.78) | 1.7 (0.80) | 0.07 |
| Utility | 0.8 (0.16) | 0.7 (0.17) | 0.7 (0.22) | 0.7 (0.19) | **<0.01** |

*Kruskal-Wallis test

*Table S7: EQ-5D scores stratified for gender*

| **Domain** | **Male (n=11)** | **Female (n=148)** | **p-value*** |
|---|---|---|---|
| MO | 1.7 (1.1) | 1.6 (0.78) | 0.9 |
| SC | 1.3 (0.65) | 1.2 (0.52) | 0.6 |
| UA | 1.6 (1.1) | 2.2 (0.88) | **0.04** |
| PD | 1.9 (1.2) | 2.6 (0.85) | **0.03** |
| AD | 1.3 (0.47) | 1.9 (0.83) | **0.02** |
| Utility | 0.8 (0.27) | 0.7 (0.19) | **0.04** |

*Mann-Whitney-Wilcoxon test

SRS-22r subgroup analyses

*Table S8: SRS-22r scores per age subgroup*

| **Domain** | **11-18 yrs (n=37)** | **19-29 yrs (n=41)** | **30-49 yrs (n=30)** | **50+ (n=57)** | **p-value*** |
|---|---|---|---|---|---|
| Function | 4.4 (0.6) | 4.1 (0.7) | 3.5 (0.7) | 3.5 (0.7) | **<0.01** |
| Pain | 3.4 (0.5) | 3.0 (0.6) | 2.8 (0.6) | 2.8 (0.6) | **<0.01** |
| Mental health | 3.8 (0.7) | 3.5 (0.7) | 3.5 (0.8) | 3.6 (0.6) | 0.1 |
| Self-image | 3.6 (0.9) | 3.6 (0.7) | 3.4 (0.6) | 3.1 (0.7) | **0.01** |
| Management (dis)satisfaction | 2.9 (0.4) | 3.1 (0.5) | 3.0 (0.5) | 3.0 (0.6) | 0.07 |
| All domains | 3.6 (0.4) | 3.4 (0.4) | 3.3 (0.5) | 3.2 (0.4) | **0.01** |

*Kruskal-Wallis test

*Table S9: SRS-22r scores stratified for gender*

| **Domain** | **Male (n=11)** | **Female (n=154)** | **p-value*** |
|---|---|---|---|
| Function | 4.1 (0.70) | 3.8 (0.71) | 0.2 |
| Pain | 3.6 (1.3) | 3.8 (2.8) | 0.3 |
| Mental health | 3.9 (0.47) | 3.6 (0.70) | 0.2 |
| Self-image | 3.7 (0.72) | 3.4 (0.74) | 0.2 |
| Management (dis)satisfaction | 3.0 (0.35) | 3.0 (0.52) | 0.7 |
| All domains | 3.6 (0.47) | 3.5 (0.64) | 0.3 |

*Mann-Whitney-Wilcoxon test

VIII: Syntax R sample code

**Patient characteristics**

```
#Data organisation
dat1<-read.csv("BurdenAIS_Final_PTCHAR.csv")

#Age categories
dat1$Agecat <- cut(dat1$Age, breaks=c(-Inf, 18,29,49, Inf), labels= c("11-18 years", "19-29 years",
"30-49 years", "50+ years"))

# Create a new variable with four education categories
dat1$new_edu <- ifelse(dat1$Education %in% c("Ik heb geen school of opleiding afgemaakt", "Lager
onderwijs (basisschool, speciaal onderwijs)"), "Lower level", ifelse(dat1$Education %in% c("Lager
beroepsonderwijs (huishoudschool, vbo, lbo, leao, lhno)", "Middelbaar algemeen onderwijs (mavo,
mulo, ivo of vmbo)", "Middelbaar beroepsonderwijs (mbo, mts, meao, mhno, inas of intas)",
"Voortgezet algemeen onderwijs (hbs, mms, havo, vwo, atheneum of    gymnasium)"), "Intermediate
level", ifelse(dat1$Education %in% c("Hoger beroepsonderwijs (hbo, hts, heao, hhno)",
"Universiteit"), "Higher level", "Other")))

#Descriptive statistics
library(summarytools)
print(dfSummary(dat1),method='render', silent=TRUE)
view(dfSummary(dat1))
```

**SRS-22**

```
#Data organisation
dat2<-read.csv("BurdenAIS_Final_SRS22.csv", sep = ";")

#assign label
dat2$Pain_6m <- factor(dat2$Pain_6m, levels = c("Veel pijn",  "Matig tot veel pijn", "Matige pijn",
"Een beetje pijn", "Geen pijn"), labels = c(1,2,3,4,5))

#New variables based on domains
dat2$MeanFunction <- ((dat2$Daily_activity + dat2$ADL + dat2$Housework + dat2$Financial +
dat2$ADL_relations)/5)

#Normality
shapiro.test(dat2$MeanFunction)

#Age categories
dat2$Agecat <- cut(dat2$Age, breaks=c(-Inf, 18,29,49, Inf), labels= c("11-18 years", "19-29 years",
"30-49 years", "50+ years"))

dat2 %>% group_by(dat2$Agecat) %>% summarise(dat2$MeanFunction)

kruskal.test(MeanFunction~Agecat, data= by_age)
```

**EQ-5D**

```
#Data organisation
dat3<-read.csv("BurdenAIS_Final_EQ5D.csv")

library(eq5d)
#eq5d5l(scores, country="Netherlands")
```

```
dat3$utility <- eq5d(scores= cbind(MO= dat3$MO,SC= dat3$SC,UA= dat3$UA,PD= dat3$PD,AD= dat3$AD),  type="VT", version="5L", country="Netherlands", ignore.invalid= TRUE)
```

**EQ5DY**

```
#Data organisation
dat4<-read.csv("BurdenAIS_Final_EQ5Y.csv")

#eq5dyouth (scores, country="Netherlands")

dat4$U_HS <- 1 - (0.036 * ifelse(dat4$MO == 2, dat4$MO, 0)) - (0.191 * ifelse(dat4$MO == 3, dat4$MO, 0)) - (0.028 * ifelse(dat4$SC==2, dat4$SC, 0)) - (0.139 * ifelse(dat4$SC==3, dat4$SC, 0)) - (0.058 * ifelse(dat4$UA==2, dat4$UA, 0)) - (0.211 * ifelse(dat4$UA==3, dat4$UA, 0))- (0.111 * ifelse(dat4$PD==2, dat4$PD, 0)) - (0.363 * ifelse(dat4$PD==3, dat4$PD, 0)) - (0.096 * ifelse(dat4$AD==2, dat4$AD, 0)) - (0.314 * ifelse(dat4$AD==3, dat4$AD, 0))
```

**MCQ**

```
#Data organisation
datMCQ<-read.csv("BurdenAIS_Final_MCQ.csv")

#Adding cost prices
datMCQ$GPPrice <- c("40.32")

#Descriptives
min(datMCQ$GP_B)
max(datMCQ$GP_B)
mean(datMCQ$GP_B)
sd(datMCQ$GP_B)

datMCQ$GPcost <- (datMCQ$GP_B*datMCQ$GPPrice)
mean(datMCQ$GPcost)
sd(datMCQ$GPcost)
```

**Medication**

```
#Data organisation
dat2<-read.csv("BurdenAIS_Final_MCQ_Meds.csv", sep = ";")

#Medication
dat2$Deliverycost <- c("7.33")
```

*OTC*

```
dat2$Aspirine <- c("0.22")
dat2$Medicine_dosage1 <- as.numeric(as.character(dat2$Medicine_dosage1))
dat2$Medicine_volume1 <- as.numeric(as.character(dat2$Medicine_volume1))
```

*Frequency*

```
dat2$Aspirine_frequency <- ifelse(dat2$Medicine_name1 %like% 'Aspirine',
dat2$Medicine_dosage1*dat2$Medicine_volume1*dat2$Medicine_totaldays1,
ifelse(dat2$Medicine_name2 %like% 'Aspirine',
dat2$Medicine_dosage2*dat2$Medicine_volume2*dat2$Medicine_totaldays2,
ifelse(dat2$Medicine_name3 %like% 'Aspirine',
dat2$Medicine_dosage3*dat2$Medicine_volume3*dat2$Medicine_totaldays3,
as.factor(NA)))))))))))))))
```

*Costs*
```
dat2$Aspirine_cost <- (dat2$Aspirine_frequency*dat2$Aspirine)
```

**Friction period**

```
#Data organisation
datFP<-read.csv("Frictionperiod.csv")

datFP$Frictionperiod_days <- ((365/((sum(datFP$Vervuld))/ (mean(datFP$Openstaand))))+4*7)
datFP$Frictionperiod_weeks <- (datFP$Frictionperiod_days/7)
```

**PCQ**

```
#Data organisation
datPCQ<-read.csv("BurdenAIS_Final_PCQ.csv", sep = ";")

#Adding cost prices
datPCQ$UnpaidworkPrice <- c("17.10")

#Hours worked per day
datPCQ$Hours_day <- ifelse(datPCQ$Hours_Paid > 0 & datPCQ$Workdays > 0,
(datPCQ$Hours_Paid/datPCQ$Workdays), "0")

#Descriptives
library(summarytools)
print(dfSummary(datPCQ),method='render', silent=TRUE)
view(dfSummary(datPCQ))

#Absenteeism
#Short absenteeism (max sickdays in 4 wks ==28 days or 20 workdays for a 5 day workweek)
datPCQ$ShortAb <- ifelse(datPCQ$Sick == "1" & datPCQ$Sickdays<= 28, (datPCQ$Sickdays *
datPCQ$Hours_day * datPCQ$ProductivityPrice), "0")

#Days >4wk absence
datPCQ$days_difference_between_Q_and_sick <- datPCQ$Sick4wk_workdays
datPCQ$days_difference_between_Q_and_sick[is.na(datPCQ$days_difference_between_Q_and_sick
)] <- 0

#Absence shorter than friction period (friction period =19.6 wks = 137.2 days, rounded to 138 days)
datPCQ$MediumAb <- ifelse(datPCQ$Sickdays_4wk == "1" &
datPCQ$days_difference_between_Q_and_sick< 138,
((((datPCQ$days_difference_between_Q_and_sick/7)*datPCQ$Workdays)*datPCQ$Hours_day)*dat
PCQ$ProductivityPrice), "0")

#Absence longer than friction period
datPCQ$LongAb <- ifelse(datPCQ$Sickdays_4wk == "1" &
datPCQ$days_difference_between_Q_and_sick>= 138,
(((19.6*datPCQ$Workdays)*datPCQ$Hours_day)*datPCQ$ProductivityPrice), "0")

#Presenteeism
datPCQ$Presenteeism_hours <-(datPCQ$Complaints_days *(1-
(datPCQ$Complaints_scale/10))*datPCQ$Hours_day)
datPCQ$Presenteeism_days <- (datPCQ$Presenteeism_hours/8)

#Productivity loss unpaid work
datPCQ$Productivity_volume <-
```

```
datPCQ$Unpaidwork_days=if_else(is.na(datPCQ$Unpaidwork_days), 0 ,
datPCQ$Unpaidwork_days)) * (datPCQ$Replacement_hours=if_else (is.na
(datPCQ$Replacement_hours), 0 , datPCQ$Replacement_hours))
```

**Total costs per sector**

*Healthcare sector*

```
datMCQ$totalhealthcare_costs <- (datMCQ$GPcost+ datMCQ$SWcost+ datMCQ$PT_cost+
datMCQ$OT_cost+ datMCQ$ST_cost+ datMCQ$D_cost+ etc.)
mean(datMCQ$totalhealthcare_costs)
sd(datMCQ$totalhealthcare_costs)
```

*Patient & Family sector*

```
datMCQ$patientfamilycost <- (datMCQ$Transportcosttotal + datMCQ$parkingcost +
datMCQ$OTCcosts)
mean(datMCQ$patientfamilycost)
sd(datMCQ$patientfamilycost)
```

*Other sector*

```
datMCQ$othersectorcosts <- datMCQ$Schooldays
datMCQ$othersectorcosts[is.na(datMCQ$othersectorcosts)] <-0
mean(datMCQ$othersectorcosts)
sd(datMCQ$othersectorcosts)
```

*Productivity sector*

```
datMCQ$totalproductivitycosts[is.na(datMCQ$totalproductivitycosts)] <-0
mean(datMCQ$totalproductivitycosts)
sd(datMCQ$totalproductivitycosts)
```

*TOTAL SOCIETAL COSTS*

```
datMCQ$totalsocietal_costs <- (datMCQ$totalhealthcare_costs+ datMCQ$patientfamilycost+
datMCQ$othersectorcosts+ datMCQ$totalproductivitycosts)
mean(datMCQ$totalsocietal_costs)
sd(datMCQ$totalsocietal_costs)
```

**Bootstrap**

```
# (1) Set the nr of bootstrap replications
set.seed(123456789)

BS <- 1000

# (2) create dataframe for bootstrap results
bootstraps <- data.frame(sim_nr = 0, totalhealthcare_costs12=0, patientfamilycost12=0,
othersectorcosts12=0, totalproductivitycosts12 = 0, totalsocietal_costs12=0)

for(i in 1:BS){

# Sample rows random with replacement
t <- sample(c(1:as.numeric(nrow(datMCQ))),as.numeric(nrow(datMCQ)),replace=T)

# Create a new dataset based on the resampled rows
datasetx <- datMCQ[t,]

 # Calculate mean utilities
```

```
totalhealthcare_costs12 <- mean(datasetx$totalhealthcare_costs12)
patientfamilycost12 <- mean(datasetx$patientfamilycost12)
othersectorcosts12 <- mean(datasetx$othersectorcosts12)
totalproductivitycosts12 <- mean(datasetx$totalproductivitycosts12)
totalsocietal_costs12 <- mean(datasetx$totalsocietal_costs12)

scores <- c(sim_nr = paste(i), totalhealthcare_costs12, patientfamilycost12, othersectorcosts12,
totalproductivitycosts12, totalsocietal_costs12)

# Add the dataframe to each "row" of the bootstrap data.frame (each "i)
bootstraps[i,] <- scores } # this ends the bootstrap loop

# (3) Show Bootstrapped results

cols <- names(bootstraps)
# Set results of bootstrap to "numeric" (as they are numbers)
bootstraps[cols] <- lapply(bootstraps[cols], as.numeric)

# Get means of all created bootstrapped variables
summary(bootstraps)
```
